# 4559 TX TM: Formalization and Institutional Investment in a Model Designed to Advance Research Translational to Community Transformation

**DOI:** 10.1017/cts.2020.363

**Published:** 2020-07-29

**Authors:** Tabia Henry Akintobi, Tabia Henry Akintobi, Breana Blaess, Brittaney Bethea, Virginia Floyd, David Hefner, Shelia McClure, Vincent Bond, Sandra Harris Hooker, Herman Taylor

**Affiliations:** 1 Morehouse School of Medicine; 2 Emory University

## Abstract

OBJECTIVES/GOALS: Morehouse School of Medicine (MSM), T^xTM^ is a scientific philosophy promoting interdisciplinary approaches towards exponential advances in community and population health. Objectives are to detail the model, pilot funding mechanism, early research findings and infrastructure investments. METHODS/STUDY POPULATION: The health research system has widely acknowledged challenges that can delay research translation to systems that advance health for chronically disadvantaged health disparity population groups. MSM’s vision is to lead the creation and advancement of health equity. The vision-aligned strategic plan prioritized formalization of a T^X TM^ implementation priority. The study population was the institution’s research faculty and leaders, research administration, and communication arm. Through a cross-institutional working group, a plan was deployed to 1) assess the institutional landscape, 2) review the grey and peer reviewed literature on translational research and 3) invest in a pilot research funding mechanism. RESULTS/ANTICIPATED RESULTS: Over $700K has been invested in T^X TM^ implementation. Over half of research faculty completed an institutional landscape assessment to identify translational research expertise, interests and points of interest in new collaboration. The most frequently cited collaborative research interests were clinical research with human subjects, patient-centered outcomes and laboratory-based research with human subjects/specimens. Funded multidisciplinary and/or community-engaged pilot studies investigate the role for circadian rhythms and shift work, cultural variables influencing mental health among Haitians living in the US and integrating prescription reconciliation telehealth in primary care. DISCUSSION/SIGNIFICANCE OF IMPACT: T^X TM^ requires interdisciplinary collaboration across translational research spheres *and* beyond the academy. Institutional investment, infrastructure support and senior-level champions are central to awareness and rewarding such scholarship towards scaling approaches that advance health equity. CONFLICT OF INTEREST DESCRIPTION: Coined at Morehouse School of Medicine (MSM), T^x TM^ symbolizes an approach and scientific philosophy designed to intentionally promote and support convergence of interdisciplinary approaches and scientists to stimulate exponential advances for the health of diverse communities.

